# Preventive Effects of Curcumin Against Drug- and Starvation-Induced Gastric Erosions in Rats

**DOI:** 10.3797/scipharm.1207-17

**Published:** 2013-01-07

**Authors:** Saida Haider, Fizza Naqvi, Saiqa Tabassum, Sadia Saleem, Zehra Batool, Sadia Sadir, Sumaira Rasheed, Darakhshan Saleem, Amber Nawaz, Saara Ahmad

**Affiliations:** 1 Neurochemistry and Biochemical, Neuropharmacological Research Unit, Department of Biochemistry, University of Karachi, Karachi-75270, Pakistan.; 2 Sir Syed University of Engineering & Technology, Karachi-75300, Pakistan.; 3 Baqai Medical University, Karachi, Pakistan.

**Keywords:** Curcumin, Antipyretic, Gastroprotective, Antinociceptive, Acetylsalicylic acid

## Abstract

The present study was designed to investigate the gastroprotective, analgesic, and antipyretic effects of curcumin (Cur), the major constituent of turmeric. Acetylsalicylic acid (ASA) was used in this study as a standard drug for comparison. The analgesic activity was measured using the Hot-Plate Test. The antipyretic and antiulcer effects were assessed using yeast-induced pyrexia and gastric ulceration, respectively. Curcumin (100 mg/kg) injected intra-peritoneally 1 hr prior to the Hot-Plate Test showed significant analgesic activity expressed by both parameters: an increase in latency time and a reduction in paw licking as compared to the controls. In the animal model of pyrexia, curcumin (100 mg/kg injected intra-peritoneally) exhibited a significant reduction in the rectal temperature after 1 hr, 2 hrs, 4 hrs, and 5 hrs of treatment, indicating the antipyretic effect of curcumin. Rats with orally administered curcumin (200 mg/kg) did not show any lesions on the inner lining of the stomach after a 16 hr fast, indicating the gastroprotective effects of curcumin as compared to saline- and acetylsalicylic acid-administered rats. The significantly low ulcer index in curcumin-treated rats following starvation highlights the gastroprotective characteristics of curcumin.

## Introduction

Curcumin (diferuloyl methane) is a phytochemical which is the active ingredient in turmeric and constitutes about 3–4% of it, giving turmeric its characteristic radiant yellow color [1, 2]. Curcumin comprises three tautomeric forms i.e. Curcumin I (curcumin) (94%), Curcumin II (demethoxycurcumin) (6%), and Curcumin III (bis-demethyoxy curcumin) (0.3%), out of which its enol-tautomeric form (Curcumin I) is more common and active [1, 3, 4]. It was first isolated in 1815 and its chemical structure was determined in 1973. Its melting point ranges from 176–177°C, forms a reddish brown salt with alkali, and is soluble in ethanol, chloroform, and alkali [1]. Curcumin is a lipophilic molecule [4] and can cross the blood-brain barrier [5]. It is well-tolerated and does not appear to be toxic even at high doses in both humans and animals [4, 6, 7]. Pharmacokinetic studies have shown that 40–85% of an oral dose of curcumin passes through the GIT unchanged [8, 9].

Curcumin possesses a broad spectrum of biological actions including anti-inflammatory, anti-carcinogenic, anti-mutagenic, anti-coagulant, anti-diabetic, anti-bacterial, anti-venom, anti-fertility, anti-protozoal, anti-fibrotic, anti-oxidant, hypocholesteremic, anti-aging, and anti-lipofusinogenesic properties [1, 2, 4]. The antiulcer activity of curcumin was displayed by attenuation of different ulcerative effectors including gastric acid hypersecretion, total peroxides, myeloperoxidase (MPO) activity, interleukin-6 (IL-6), and apoptotic incidence, along with its inhibitor activity for pepsin [10]. Inhibitory effects of curcumin on lipoxygenases, cyclooxygenase isoenzymes, and inflammatory cytokine production have also been reported [11]. Curcumin also offers protection against vascular dementia by exerting antioxidant activity [1, 12].

The present study was designed to evaluate the gastroprotective effects of curcumin. The study was also aimed at investigating the analgesic and antipyretic effects of curcumin. Since acetylsalicylic acid is one of the commonly used drugs with a broad spectrum of pharmacological activities, in the present study it was taken as a standard drug to compare the effects of curcumin.

## Results

**Fig. 1:**
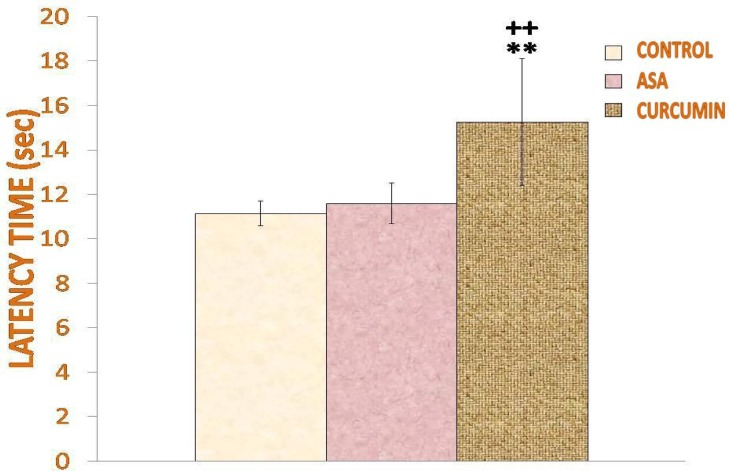
Effect of curcumin on latency time in the Hot-Plate test. Values are expressed as mean + SD and significant differences by Newman-Keuls test following 1-way ANOVA are * = p<0.05, ** = p<0.01 as compared to saline-injected rats and + = p<0.05, ++ = p<0.01 as compared to acetylsalicylic acid-injected rats

**Fig. 2:**
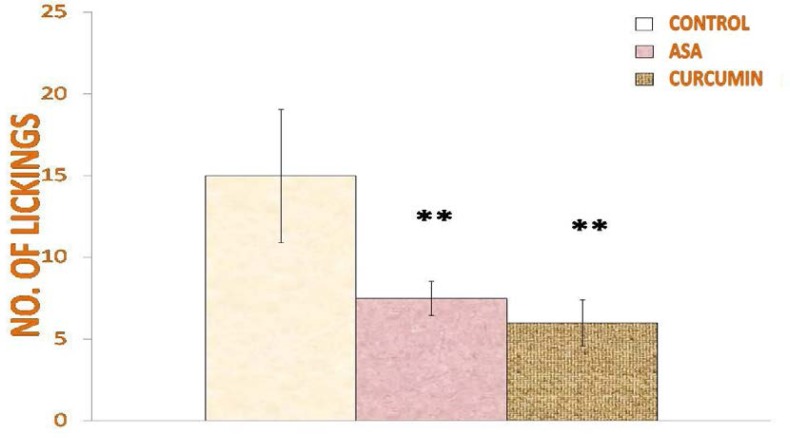
Effect of curcumin on number of lickings in the Hot-Plate test. Values are expressed as mean + SD and significant differences by Newman-Keuls test following 1-way ANOVA are * = p<0.05, ** = p<0.01 as compared to saline-injected rats and + = p<0.05, ++ = p<0.01 as compared to acetylsalicylic acid-injected rats

Anti-nociceptive tests showed that acute treatment with curcumin increases the reaction time in response to thermal pain (Fig. 1). Figure 1 shows that curcumin-treated rats exhibited greater (p<0.01) latency time (15.26±2.85 sec) as compared to those treated with aspirin (11.6±0.909 sec) and saline (11.15±0.568 sec). The number of lickings of both curcumin- and acetylsalicylic acid-treated rats were significantly lower (p< 0.01) than that of the saline-treated rats (Fig. 2). The number of lickings for curcumin was lower than that of acetylsalicylic acid (7.5±1.290) which was, however, not statistically significant.

**Fig. 3:**
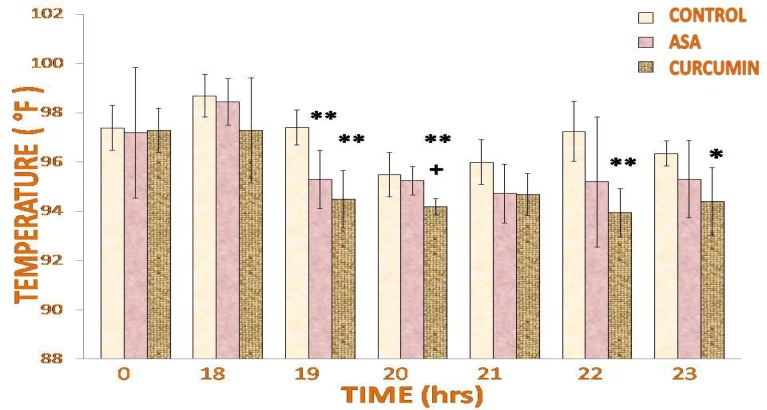
Effect of curcumin on pyrexia induced by yeast suspension. Rectal temperature determination showed curcumin significantly lowers the temperature after treatment. Values are expressed as mean + SD and significant differences by Newman-Keuls test following 1-way ANOVA are * = p<0.05, ** = p<0.01 as compared to saline-injected rats and + = p<0.05, ++ = p<0.01 as compared to acetylsalicylic acid-injected rats

Eighteen hours after the injection of the yeast suspension (pyrogen), fever had developed. Treatment with curcumin significantly lowered the temperature when compared with saline. At 1 hour post-treatment, the temperatures of both curcumin-treated (94.5±1.16°F) and acetylsalicylic acid-treated rats (95.3±1.188°F) were significantly (p<0.01) lower than that of the saline-treated rats (97.42±0.71°F). After 2 hours of treatment, curcumin (94.2±0.33°F) reduced the rectal temperatures significantly (p<0.05) as compared to the acetylsalicylic acid-treated rats (95.25±0.575°F) and (p<0.01) saline-treated rats (95.5±0.899°F). But at 4 and 5 hours post-treatment, the drop in rectal temperatures was significant (p<0.01, p<0.05; respectively) only in the curcumin-treated rats (Fig. 3).

**Fig. 4:**
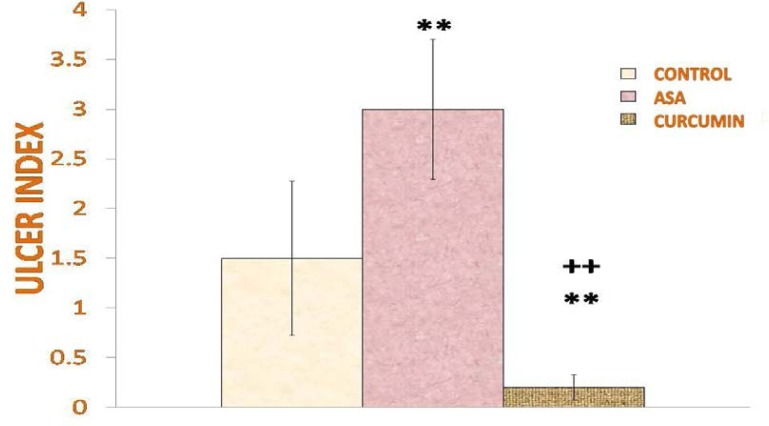
Effect of curcumin and acetylsalicylic acid on stomach ulcer induced by 16 hours fasting represented in terms of ulcer index. Ulcer index showed that curcumin significantly (p<0.01) reduces the number of gastric lesions as compared to control and acetylsalicylic acid-treated rats. Values are expressed as mean + SD and significant differences by Newman-Keuls test following 1-way ANOVA are **=p<0.01 as compared to saline-injected rats and ++ = p<0.01 as compared to acetylsalicylic acid-injected rats

Rats administered with acetylsalicylic acid showed more prominent gastric lesions on the internal lining of the stomach as compared to the stomachs treated with saline, which had fewer lesions, along with curcumin. Scoring of gastric mucosal lesions revealed that the ulcer index was significantly higher in acetylsalicylic acid-treated rats as compared to saline- (p<0.01) and curcumin- (p<0.01) administered rats (Fig 4), whereas rats administered with curcumin exhibited a low ulcer index (p<0.01) compared to saline- and acetylsalicylic acid-treated rats (Fig. 4).

## Discussion

Curcumin has been used for centuries as a treatment for inflammatory diseases with a long history of medicinal use in India [13]. Since inflammation is mostly involved in various psychopathological conditions including pain (nociception) and fever (pyrexia), the investigation of the analgesic and antipyretic potential of curcumin and its comparison with one of the most widely used drugs, acetylsalicylic acid, has been targeted in this study. In the present investigation, the analgesic effect of curcumin was determined by the Hot-Plate method. This method is a widely used model for the evaluation of pain. The Hot-Plate test measures complex responses to acute nociceptive exposure [14]. In this study, curcumin significantly reduced the response against the thermal stimulus indicated by an increase in the latency time and a decrease in the paw licking, reflecting the analgesic activity of the drug. Prostaglandins (PGs) are known to sensitize the pain receptors at the local site of the stimulus [15]. PGs are synthesized from arachidonic acid via the cyclooxygenase and lipoxgenase pathways. Thus, suppression of these enzymes (cyclooxygenase-2 and 5-lipoxygenase) by curcumin as reported earlier [16] inhibits prostaglandin synthesis and may be attributed to its pain relieving action. PGs are considered to act in the spinal cord to facilitate the transmission of pain responses [17], so inhibition of this pathway by curcumin may be yet another possible mechanism.

The antipyretic effect of curcumin was evaluated by an animal model of pyrexia in which fever was induced by a yeast suspension, a non-toxic pyrogen. This method has been used in various studies to monitor pyrexia after yeast administration and to evaluate the activity of antipyretic drugs after treatment [18, 19]. Fever is produced in response to a number of factors; however, it is an adaptive mechanism for controlling infection. In most cases, the cause is viral although bacterial infections and inflammatory diseases need early diagnostic considerations. Synthesis of endogenous pyrogens (prostaglandins) in response to a stimulus signals the thermoregulatory center of the brain and results in an elevation of body temperature. Curcumin showed a significant antipyretic effect by decreasing the rectal temperature in our study. It has been reported earlier that curcumin has an inhibitory impact on prostaglandins [4] and hence, our findings are in agreement with the previous findings.

Previously, starvation has been shown to aggravate drug-induced gastric erosions [20, 21]. In the present study, administration of acetylsalicylic acid on an empty stomach has shown severe gastric lesions and serious damage to the internal lining of the gastric mucosa. After opening and washing the stomachs of acetylsalicylic acid-treated rats, it was observed that the gastric mucosal layer appeared reddish and also showed swelling. Saline-treated rats also exhibited a slightly reddish mucosal lining. However, rats administered with curcumin in the fasting condition did not show reddish lesions or swelling of the gastric mucosa. Use of NSAIDs, including acetylsalicylic acid, is the most common cause of gastrointestinal mucosal injury. Severe gastrointestinal injuries and lesions due to NSAIDs, such as acetylsalicylic acid, are mainly caused by the inhibition of COX-1 [22]. It has been reported that COX-2 mostly leads to the production of PGE_2_ which plays an important role in inflammatory reactions, whereas COX-1 causes the production of prostaglandins which regulate physiological process including protection of the stomach mucosa [23]. Curcumin has the potential to act against ulcers in the stomach. Not much work regarding the effects of curcumin in gastric ulcers is reported, however in one study, the beneficial effect of curcuma longa has been reported due to its selective as well as competitive antagonistic activity towards the H_2_ receptor located on gastric parietal cells [24].

## Conclusions

In conclusion, our findings of a significantly reduced ulcer index in curcumin-treated rats provide evidence that curcumin is not only effective as an anti-nociceptive and anti-pyretic agent, but it also possesses gastroprotective activity. The gastroprotective effects of curcumin observed in the present study may therefore suggest the pharmacological advantage of curcumin over commercially available NSAIDs to prevent gastric ulcers. However, further experiments are warranted to determine the actual mechanism of action of curcumin.

## Experimental

Experimental animals were all healthy adult female Sprague Dawley rats weighing (150– 180g). Fifty four locally bred female rats purchased from Agha Khan University were used in this study. All animals were housed individually under a 12hr light-dark cycle (light on at 6:00 h) and controlled room temperature (22±2°C) with free access to cubes of standard rodent diet and tap water for at least 3–4 days before experimentation so that rodents could adapt themselves to the new environment. All animal experiments were conducted in accordance with NIH guidelines and approved by the institutional Ethics and Animal Care Committee. Animals were randomly divided into three groups each containing 18 rats. Each set of 18 rats was further divided into three sub-groups: Control, acetylsalicylic acid (ASA), and curcumin (Cur). Animals in the control group were injected with 0.9% saline, the second group of animals were injected with acetylsalicylic acid (10mg/kg), and the third group with curcumin (100mg/kg) intra-peritoneally [25] and the following tests were performed.

### Anti-nociceptive Test

For evaluation of the analgesic effects, animals were injected with saline, acetylsalicylic acid, and curcumin intra-peritoneally and 30 minutes post-injection, the Hot-Plate test was performed. The method was essentially the same as described by Eddy and Leimbach, 1953 [14]. It is a test of pain response in animals. It is used in testing the effectiveness of analgesics by observing the reaction to pain caused by heat. The temperature of the plate is set at 55°C and is kept constant. The rat is placed on the plate and is covered with a cylinder so that the animal is unable to move out. The analgesic activity is observed in terms of latency to move and forepaw licking. The total cut-off time is 3 minutes or 180 seconds. If the administered drug induces analgesic effects, then the latency to move increases and the forepaw licking decreases showing the pain-reducing effect of the drug.

### Antipyretic Test

The rats were injected subcutaneously with 10 ml/kg of a 20% aqueous suspension of Baker’s yeast and the rectal temperatures were recorded initially and at 18 hours. Saline, acetylsalicylic acid (10mg/kg), and curcumin (100mg/kg) were administered intra-peritoneally to the control, ASA, and Cur groups, respectively after 18 hrs reading. Following the administration of drugs, rectal temperature was recorded up to 5h as per method of Mukherjee et al. 1996 [26].

### Ulcerogenic Test

Following 16 hours of fasting, animals in the Control, ASA, and Cur groups were orally given 1 ml of neutral oil, acetylsalicylic acid (20 mg/kg), and Cur (200 mg/kg), respectively. After three hours of administration, the animals were killed and their stomachs were removed. The stomachs were opened by placing a cut along the lesser curvature and washed with saline in order to visualize the internal lining of the stomach. The gastric mucosa was then scored according to the scale, 0: no lesion, 0.5: hyperemia, 1: one or two lesions, 2: severe lesions, 3: very severe lesions, 4: mucosa full of lesions [27, 28].

### Drugs

The commercially available curcumin (Color: orange, M. wt: 368.38, Form: powder) and acetylsalicylic acid (Color: white, M. wt.: 180.16, Form: Powder) were purchased from Sigma Aldrich. All chemicals were of analytical grade. All the reagents were freshly prepared before the start of the experiment. For the oral administration, the curcumin suspension was prepared in cooking oil, whereas the intra-peritoneal administration curcumin suspension was prepared in water and ethanol in the ratio of 9:1. Acetylsalicylic acid was dissolved in deionized water.

### Statistical Analysis

Data are expressed as the mean ± S.D and were analyzed by One Way ANOVA followed by Newman’s Keul test. Values of p<0.05 were regarded as significant.
